# Successful Pre-Treatment Ovarian Fresh Tissue Transplantation in a Cervical Cancer Patient Undergoing Radiation Therapy: A Case Report

**DOI:** 10.7759/cureus.43472

**Published:** 2023-08-14

**Authors:** Marília A. Bertolazzi, Maria Luiza Nogueira Dias Genta, Filomena Carvalho, Edmund C. Baracat, Jesus Paula Carvalho

**Affiliations:** 1 Department of Gynecologic Oncology, Universidade de São Paulo, São Paulo, BRA; 2 Department of Pathology, Faculdade de Medicina da Universidade de Sao Paulo, Sao Paulo, BRA; 3 Department of Gynecology, Universidade de São Paulo, São Paulo, BRA; 4 Department of Gynecologic Oncology, Hospital Sirio Libanes, Sao Paulo, BRA; 5 Division of Gynecologic Oncology, Department of Obstetrics and Gynecology, Instituto do Cancer do Estado de Sao Paulo (ICESP/HC/FMUSP) Faculdade de Medicina. Universidade de Sao Paulo, Sao Paulo, BRA

**Keywords:** cancer radiotherapy, pelvic radiotherapy, hormonal therapy, premature ovarian insufficiency, uterine cervical cancer

## Abstract

Cervical cancer is one of the most frequent gynecological malignancies in Brazil, and most of the patients require pelvic radiotherapy as part of oncological treatment.

Pelvic radiotherapy induces ovarian premature insufficiency in pre-menopausal women. This condition impacts the life quality and increases the risk of osteoporosis, obesity, cardiovascular, and neurodegenerative diseases in the middle and long term.

Most of these patients have no access to hormonal replacement therapy. Techniques such as ovarian transposition have questionable results when aiming to preserve ovarian function. In this context, a promising alternative is the implantation of fresh ovarian tissue, outside the radiotherapy field, in the abdominal cavity (orthotopic implantation) or in other sites such as the forearm, breast, or subcutaneous tissue (heterotopic implantation).

Here we report a successful case of autologous implantation of fresh ovarian tissue in the inner thigh of a young patient with advanced cervical cancer, who was a candidate for concurrent chemoradiotherapy.

## Introduction

Cervical cancer is one of the most frequent gynecological malignancies in Brazil, with an incidence of more than 16,000 cases per year [1]. Three-quarters of the patients are diagnosed in the advanced stages (beyond stage IB2 - FIGO [International Federation of Gynecology and Obstetrics] 2018) [2]. Early stage tumors are properly treated with surgery, while locally advanced tumors can be treated with a combination of surgery and radiotherapy or radiotherapy with concurrent chemotherapy. In our institution, of 292 cervical cancer patients treated between 2008 and 2012, 82.9% had indication of radiotherapy as part of the treatment [2].

The ovaries are sensitive to ionizing radiation. Doses such as 250-300 cGy can suppress ovarian function [3]. Radiotherapy for cervical cancer curative treatment induces irreversible ovarian insufficiency and harms the reproductive and hormonal functions, even in regimens with reduced radiation doses or planning based on intensity-modulated radiotherapy.

Ovarian premature insufficiency has severe consequences in life quality, such as climacteric and genitourinary symptoms, sexual dysfunction, and increased risk of osteoporosis, obesity, cardiovascular, and neurodegenerative diseases in the middle and long term [4]. Young cervical cancer survivors treated with radiotherapy are the most impacted in terms of life quality when compared to other types of gynecological cancer survivors [5,6].

Ovarian transposition is a technique with questionable results when aiming to preserve ovarian function. Less than 40% of women maintain ovarian function for a long term when submitted to ovarian transposition before radiation therapy [7]. In this context, a promising alternative is the implantation of fresh ovarian tissue, outside the radiotherapy field, in the abdominal cavity (orthotopic implantation) or in other sites such as the forearm, breast, or subcutaneous tissue (heterotopic implantation) [8,9].

Here we report a successful case of autologous implantation of fresh ovarian tissue in the inner thigh of a young patient with advanced cervical cancer, candidate for concurrent chemoradiotherapy.

## Case presentation

A 26-year-old female patient with squamous cell carcinoma of the uterine cervix, FIGO stage IIIC1, underwent the procedure for autologous implantation of fresh ovarian tissue one week before the start of oncological treatment. Serum levels of follicle-stimulating hormone (FSH) and estradiol before the procedure were 4.8 mUI/mL and 225 ng/dL, respectively.

**Figure 1:**
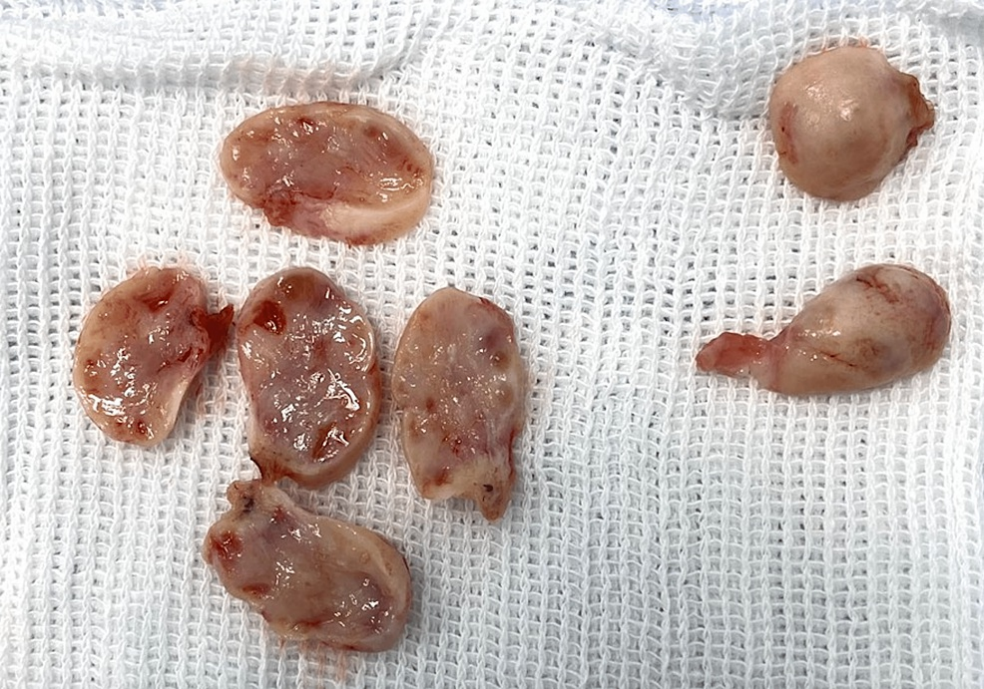
Ovarian slices

The procedure began with laparoscopic unilateral right-side oophorectomy. We removed the ovary protected by endobag and then sectioned it into 1- to 2-mm-thick slices (Figure 1). One of the slices underwent anatomopathological examination.

**Figure 2:**
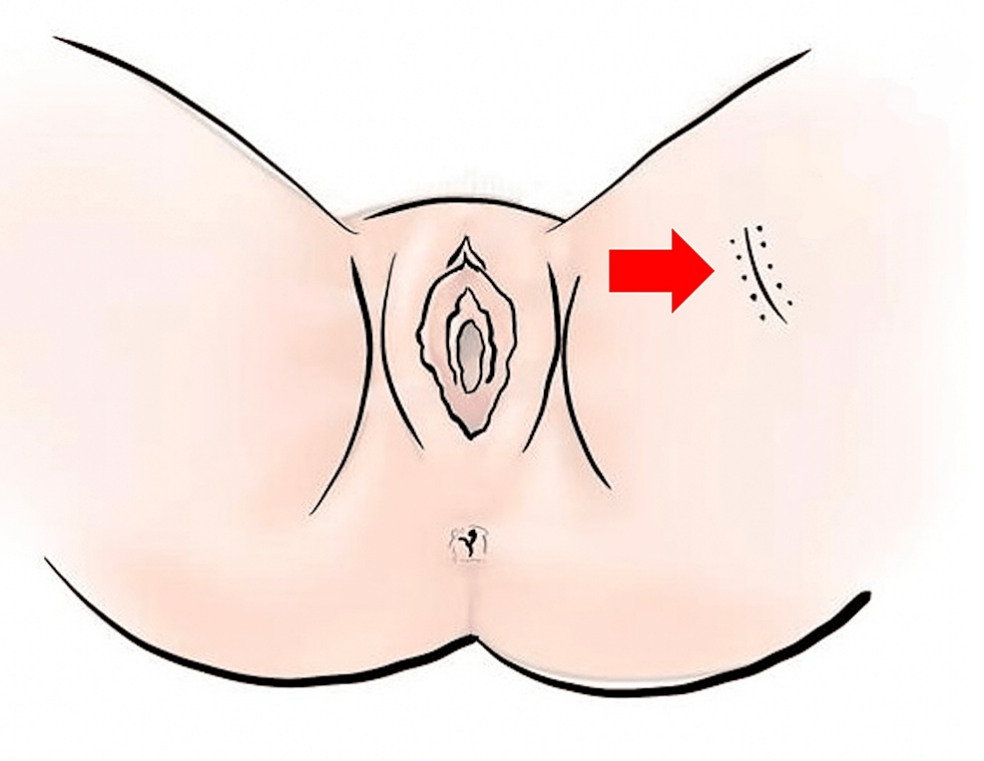
Location of the implant in the inner thigh

**Figure 3:**
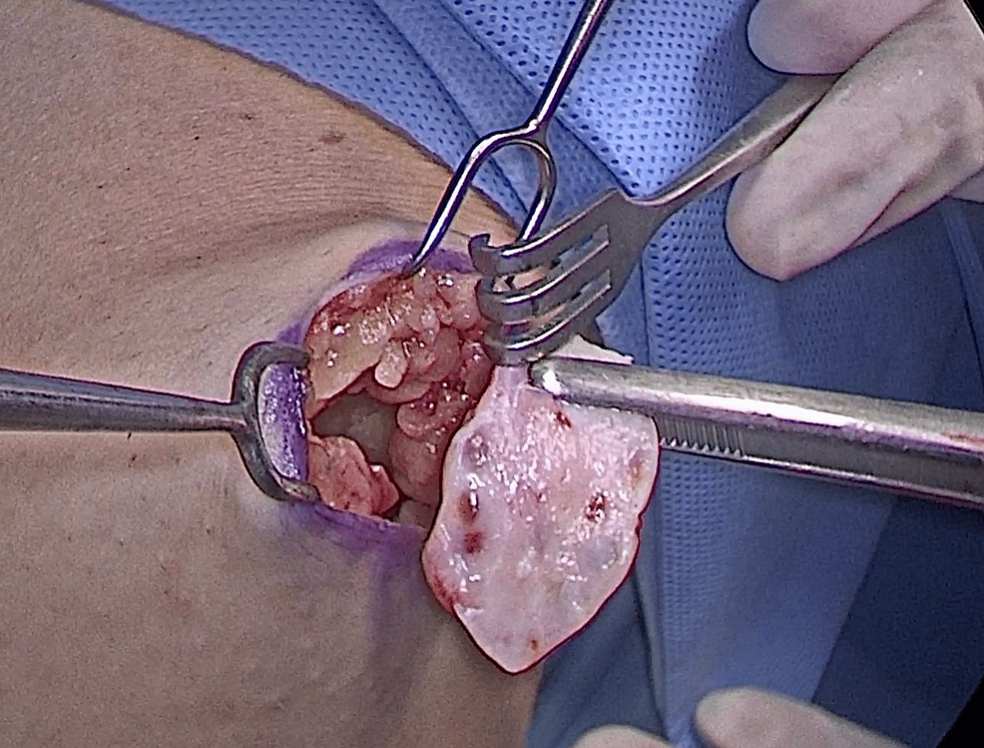
Placing ovarian graft in the deep subcutaneous tissue

Through a horizontal incision of 2 cm on the inner face of the thigh (Figure 2), we created a space in the deep subcutaneous tissue below Scarpa's fascia and above the muscular plane. We placed five slices of ovarian tissue into this site and closed the adjacent subcutaneous and skin (Figure 3). We performed ultrasound examinations to assess the viability of the ovarian tissue. One week later, the patient began oncologic treatment, which included 25 sessions of pelvic radiotherapy with a total dose of 5,580 Gy associated with concomitant chemotherapy with cisplatin and four sessions of brachytherapy with a total dose of 2,800 Gy.

**Figure 4:**
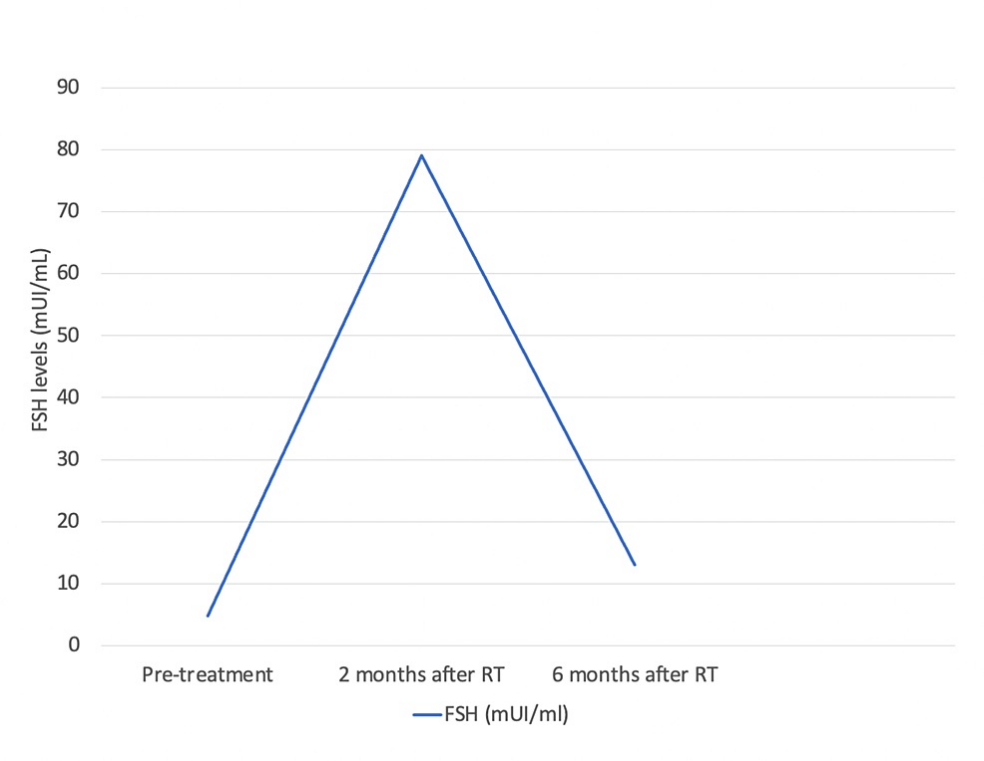
FSH levels pre-treatment and at two and six months after radiotherapy FSH, follicle-stimulating hormone

**Figure 5:**
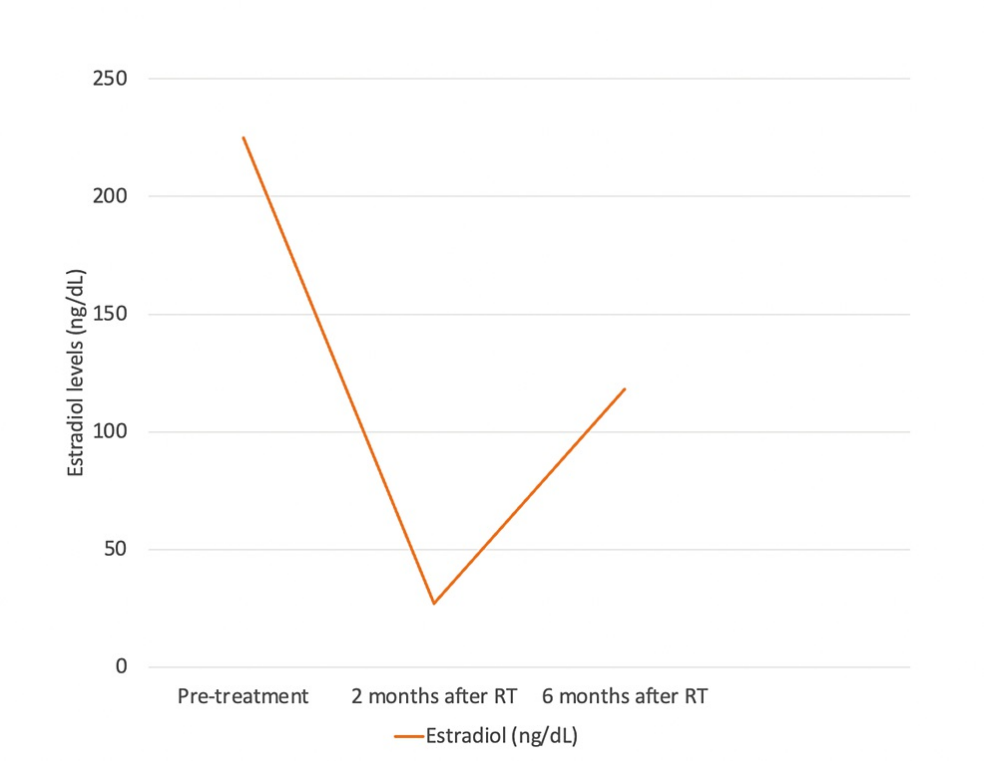
Estradiol levels pre-treatment and at two and six months after radiotherapy

Two months after the end of radiotherapy, the patient presented hot flashes, irritability, and anxiety symptoms. The serum FSH level increased to 79 mUI/mL. Four months after the end of radiotherapy and six months after implantation, the patient presented resolution of climacteric symptoms and normalization of FSH and estradiol levels. Six months after the end of radiotherapy, the serum levels of FSH and estradiol were 13 mIU/mL and 118 ng/dL, respectively. The reference value of FSH for premenopausal women is under 25 mUI/mL. Estradiol levels in menopausal women are under 28 ng/dL. The levels of FSH and estradiol pre-treatment and at two and six months after radiotherapy are summarized in Figures 4, 5.

## Discussion

Several studies have shown that a minority of cervical cancer survivors receive adequate hormonal therapy. Only 13% of these patients receive a formal diagnosis of ovarian premature insufficiency and less than 40% receive some hormone replacement prescription. General practitioners distrust prescribing hormonal therapy and face challenges as a lack of medication in the public health system [10-13].

In this context, despite the potential to circumvent the problem in this group of patients, hormonal replacement is rarely maintained in the long term [6,11,12].

Preservation of the ovaries is possible in almost all cervical cancers, even in advanced stages. Squamous cell carcinoma, which accounts for more than 80% of these tumors, spreads by contiguity to the parametrium, pelvic, and aortic lymph nodes, and, as a rule, does not compromise the ovaries. The risk of ovarian metastases is shallow, and any impairment can be diagnosed during the intra-operative examination [14-17].

Removal of the ovaries outside the irradiation field (ovarian transposition) has been performed over time, with success rates ranging from 20% to 100% depending on the primary site and the type of tumor [18,19]. In this approach, the gonads and their vascular supply are fixed in a site behind or lateral to the uterus, above the iliac crest. However, it is difficult to guarantee that the gonads are outside the irradiation field, as it can vary according to the disease staging. Lv et al. [20] reported that in a series of 214 patients whose ovaries were transposed, only 32 (15%) were located outside the planned field of radiotherapy. Radiotherapy in cervical cancer is used in combination with cisplatin. Cisplatin, used at a lower than the therapeutic dose, has a radiosensitizing effect and, at these doses, is not considered deleterious to the germ cell [21].

To maintain the ovarian hormone function in the long term, in women with no desire or no conditions to preserve fertility, the implantation of fresh ovarian tissue seems to be a simple, low-cost, and feasible procedure. This is the first successful case report of autologous implantation of fresh ovarian tissue in a young woman with advanced cervical carcinoma. This procedure, however, needs a randomized trial to be validated as an alternative to ovarian transposition or hormone replacement therapy. A pilot study is underway at our service with a series of 20 cases.

## Conclusions

Autologous fresh ovarian tissue transplantation to the subcutaneous tissue of the inner tight could be an acceptable alternative for maintaining ovarian hormone function in young patients with cervical cancer, who are candidates for treatment with pelvic radiotherapy. These women rarely maintain hormonal therapy after treatment for different reasons and are more exposed to side effects and repercussions of ovarian premature insufficiency. This procedure has several advantages over the ovarian transposition techniques, such as the safe distance of the transplanted ovarian tissue from the radiotherapy field, the readiness for follow-up of the graft with Doppler ultrasound, and the maintenance of hormone production in the long term. These characteristics make the technique relevant in clinical practice because cervical cancer affects predominantly low-income women. Autologous fresh ovarian tissue transplantation is a pilot study to evaluate the visibility of autologous fresh ovarian tissue transplantation in young patients. A randomized clinical trial is in course to evaluate this procedure in a cohort of patients.
